# Supplementary material for: Content-rich biological network constructed by mining PubMed abstracts
Source: BMC Bioinformatics. 2004 Oct 8;5:147. doi: 10.1186/1471-2105-5-147 (PMC528731; doi:10.1186/1471-2105-5-147)
Supplement: Additional File 2 — The original results of the above study (non-essential files are deleted to keep the file size under the limit set by BMC bioinformatics). [file 1471-2105-5-147-S2.bz2 › chilibotAdditionalFile2/dip40/110ID8604139E454/html/FLIM_FLIN.html]

 


 **FLIM** and **FLIN** 
  
Found 43 abstracts in PubMed, retrieved 40.  
 

 What does Google say? 
 PDF only 
| .edu only 

---

**Interactive relationship** (e.g. stimulation, inhibition, etc)

**Neutral relationship**- Using the two hybrid system in Saccharomyces cerevisiae, we demonstrated strong interactions between FliG  **FliM** ,  **FliM**   **FliM** , and  **FliM**   **FliN** .  Ref: 8604139 J Mol Biol, 1996
- Here, we have used affinity blotting to obtain direct evidence of interaction between Salmonella typhimurium  **FliM**  and  **FliN** ,  **FliM**  and FliG, and  **FliM**  and CheY P.  Ref: 9356251 J Mol Biol, 1997
- The results show that FliG,  **FliM**  and  **FliN**  all bind to each other,.  Ref: 8757288 J Mol Biol, 1996
- These proteins interact in the Saccharomyces cerevisiae two hybrid system FliG interacts with  **FliM** ,  **FliM**  interacts with itself, and  **FliM**  interacts with  **FliN** .  Ref: 8631704 J Bacteriol, 1996
- Distinct regions of bacterial flagellar switch protein  **FliM**  interact with FliG,  **FliN**  and CheY.  Ref: 9356251 J Mol Biol, 1997
- Two other proteins,  **FliM**  and  **FliN** , are known to bind to FliG and have also been suggested to be involved to some extent in torque generation.  Ref: 9573160 J Bacteriol, 1998
- Regulated underexpression and overexpression of the  **FliN**  protein of Escherichia coli and evidence for an interaction between  **FliN**  and  **FliM**  in the flagellar motor.  Ref: 7768859 J Bacteriol, 1995
- Complementation analysis of this  **fliM**  mutant indicated that  **fliM**  and  **fliN**  transcription starts beyond the 5 end of fliK and terminates after  **fliN** .  Ref: 9683497 J Bacteriol, 1998
- Genetic analyses and binding studies have shown that  **FliM**  interacts with several other flagellar proteins, including  **FliN** , FliG, phosphorylated CheY, other copies of  **FliM** , and possibly MotA and FliF.  Ref: 9791106 J Bacteriol, 1998
- The cytoplasmic C ring of the structure, which is seen consistently in electron microscopy of wild type complexes and which is known to contain the  **FliM**  and  **FliN**  proteins, was much more labile in the  **FliM**   **FliN**  fusion mutant, giving a fragmented and variable appearance or being completely absent.  Ref: 8755888 J Bacteriol, 1996
- Overexpression of  **FliN**  impairs motility and flagellation, as has been reported previously for  **FliM** , but when  **FliN**  and  **FliM**  are co overexpressed, motility is much less impaired.  Ref: 7768859 J Bacteriol, 1995
- One such pseudorevertant was found to contain a compensating frameshift such that the  **fliM**  and  **fliN**  genes were placed in frame, coding for an essentially complete  **FliM**   **FliN**  protein fusion.  Ref: 8755888 J Bacteriol, 1996
- We interpret these data to mean that the  **FliM**   **FliN**  fusion protein incorporates into structure but less stably than do the  **FliM**  and  **FliN**  proteins separately, that wild type  **FliM**  tends to displace the fusion protein,.  Ref: 8755888 J Bacteriol, 1996
- The  **fliN**  gene is flanked on its 5 end by the 3 end of a homolog of a  **fliM**  gene.  Ref: 7622217 Infect Immun, 1995
- The Salmonella typhimurium  **FliN**  protein has been proposed to form a mutually interacting complex with FliG and  **FliM** , the switch complex, that is required for flagellar morphogenesis and function.  Ref: 8757287 J Mol Biol, 1996
- Complementation data indicated that wild type  **FliM**  had a mild dominant negative effect over the fusion protein, that wild type  **FliN**  and the fusion protein work much better than the fusion protein alone,.  Ref: 8755888 J Bacteriol, 1996

**Non-interactive relationship** (e.g. studied together, co-existance, homology, etc.)

- The genes identified include flbD, motA, motB, fliL,  **fliM** ,  **fliN** , fliZ, fliP, fliQ, fliR, flhB, flhA, flhF and flbE.  Ref: 9168127 Gene, 1997
- Torque generation in the flagellar motor of Escherichia coli evidence of a direct role for FliG but not for  **FliM**  or  **FliN** .  Ref: 8550421 J Bacteriol, 1996
- This explains why single, missense  **fliN** , fliG or  **fliM**  mutations, found in many non motile strains, can alter the belled morphology.  Ref: 8757287 J Mol Biol, 1996
- Taken together with previous genetic, physiological and ultrastructural studies, these results provide strong support for the view that FliG,  **FliM**  and  **FliN**  function together in a complex on the rotor of the flagellar motor.  Ref: 8757288 J Mol Biol, 1996
- Mutations in Salmonella typhimurium fliG,  **fliM**  and  **fliN**  give rise either to non flagellate, non motile or non chemotactic mutant bacteria.  Ref: 7650739 J Mol Biol, 1995
- Here, we examine the effects of a set of linker insertions and partial deletions in  **FliM**  on its binding to  **FliN** , FliG, CheY, and phospho CheY and on its functions in flagellar assembly and rotation.  Ref: 9791106 J Bacteriol, 1998
- FliY is partly homologous to the Escherichia coli and Salmonella typhimurium switch proteins  **FliM**  and  **FliN** .  Ref: 1447979 Mol Microbiol, 1992
- The data support, but do not prove, a model in which  **FliM**  and  **FliN**  in the wild type switch complex are stationary with respect to each other.  Ref: 8755888 J Bacteriol, 1996
- FliG does not require  **FliM**  and  **FliN**  for this association, but  **FliM**  and  **FliN**  associate cooperatively with the membrane only through FliG.  Ref: 9006037 J Bacteriol, 1997
- Three Salmonella typhimurium flagellar motor proteins, FliG,  **FliM**  and  **FliN** , are required for the switching of rotation sense.  Ref: 10788321 J Mol Biol, 2000
- To investigate the function of  **FliM**  and its interactions with FliG and  **FliN** , we engineered a series of 34  **FliM**  deletion mutant proteins, each lacking a different 10 amino acid segment.  Ref: 8955386 J Bacteriol, 1996
- Five proteins MotA, MotB, FliG,  **FliM**  and  **FliN**  may be involved in energizing flagellar rotation in Escherichia coli.  Ref: 8627625 J Mol Biol, 1996
- Three motor proteins, FliG,  **FliM** , and  **FliN** , have been implicated in this process.  Ref: 8604139 J Mol Biol, 1996
- None of the conserved acidic residues of MotA, FliG,  **FliM** , or  **FliN**  proved essential for torque generation.  Ref: 9573160 J Bacteriol, 1998
- This and additional evidence presented indicate that  **FliM**  and  **FliN**  are associated in the flagellar motor, in a structure distinct from the MotA MotB torque generators.  Ref: 7768859 J Bacteriol, 1995
- Expression in a non motile S. typhimurium  **fliN**  mutant restores motility but not chemotaxis, although expression in a non motile E. coli  **fliM**  mutant does not restore motility.  Ref: 1447979 Mol Microbiol, 1992
- Cell lysates from these strains contained the wild type complement of FliG,  **FliM**  and  **FliN** .  Ref: 8757287 J Mol Biol, 1996
- Incubation of purified basal bodies at pH 4.5 removed  **FliM**  and  **FliN**  but not FliG or FliF.  Ref: 11591685 J Bacteriol, 2001
- but the basal bodies lacked the outer, cytoplasmic C ring of the bell and were separated by sedimentation from  **FliM**  and  **FliN** .  Ref: 8757287 J Mol Biol, 1996
- The Agrobacterium tumefaciens motor gene, motA, is in a linked cluster with the flagellar switch protein genes, fliG,  **fliM**  and  **fliN** .  Ref: 9161425 Gene, 1997
- We propose a model in which the N terminal region of  **FliM**  binds to CheY P, the middle region of  **FliM**  binds to FliG, and the C terminal region binds to  **FliN** .  Ref: 9356251 J Mol Biol, 1997
- The basal body C rings, composed of the  **FliM** , FliG and  **FliN**  proteins, form the rotor of the flagellar motor 2.  Ref: 10959841 Curr Biol< MedlineTA>Curr Biol, 1997
- The motor that drives the flagellar filament of Escherichia coli contains three switch proteins FliG,  **FliM** , and  **FliN**  that together determine the direction of rotation.  Ref: 8631704 J Bacteriol, 1996
- The switch proteins, FliG,  **FliM** , and  **FliN** , isolated to purity, were not acetylated.  Ref: 1390767 Biochemistry, 1992
- FliG,  **FliM** , and  **FliN**  are three proteins of Salmonella typhimurium that affect the rotation and switching of direction of the flagellar motor.  Ref: 8423152 J Bacteriol, 1993
- The flagellar switch of Salmonella typhimurium and Escherichia coli is composed of three proteins, FliG,  **FliM** , and  **FliN** .  Ref: 8955386 J Bacteriol, 1996
- Immunoblotting of book basal body complexes from the wild type strain gave a strong signal for the three switch proteins FliG,  **FliM** , and  **FliN** .  Ref: 8755888 J Bacteriol, 1996
- In this work, we identify the switch components, FliG,  **FliM** , and  **FliN** , of the sodium type motor.  Ref: 10648530 J Bacteriol, 2000
- The FliG,  **FliM**  and  **FliN**  proteins form part of recently characterized extended flagellar basal structures, and have been postulated to form a mutually interacting structural complex.  Ref: 7650739 J Mol Biol, 1995
- Here, we present evidence that the reported mot mutations in  **fliM**  and  **fliN**  do not disrupt torque generating functions specifically but, instead, affect the incorporation of proteins into the flagellum.  Ref: 8550421 J Bacteriol, 1996
- About 25 copies of FliG are present in a large rotor mounted assembly termed the switch complex, which also contains the proteins  **FliM**  and  **FliN** .  Ref: 12093724 EMBO J, 2002
- Electron microscopic analysis indicated that polar and lateral flagellum specific FliG,  **FliM** , and  **FliN**  switch proteins are necessary for assembly of the respective flagella.  Ref: 11948156 J Bacteriol, 2002
- FliG,  **FliM** , and  **FliN**  are part of the rotor.  Ref: 7892209 Proc Natl Acad Sci U S A, 1995
- Five proteins MotA, MotB, FliG,  **FliM** , and  **FliN**  have been implicated in energizing flagellar rotation in Escherichia coli and Salmonella typhimurium.  Ref: 7892209 Proc Natl Acad Sci U S A, 1995
- Four mutants had the KmR cassette inserted in genes possibly involved in flagella motor function pflA,  **fliM**  and orf1 downstream of the  **fliN**  gene.  Ref: 10463181 Microbiology, 1999
- Antisera to Escherichia coli and Salmonella typhimurium  **FliN** ,  **FliM** , FlhB and FlhA reacted with proteins of the predicted molecular weights in cell lysates of Bb.  Ref: 9168127 Gene, 1997
- To address this question, we purified the intact switch complex constituting the switch proteins FliG,  **FliM** ,  **FliN**  and the scaffolding protein FliF in quantities sufficient for biochemical work and used it to investigate whether or not the binding of CheY to the switch complex is cooperative.  Ref: 12736245 J Biol Chem< MedlineTA>J Biol Chem, 1997
- We have developed a preparative procedure for flagellar motors that retains these proteins as evidenced by Western blots using anti FliG, anti  **FliM**  and anti  **FliN**  antibodies.  Ref: 8308888 J Mol Biol, 1994
- The flagellar switch genes  **fliM**  and  **fliN**  of Rhodobacter sphaeroides are contained in a large flagellar gene cluster.  Ref: 9683497 J Bacteriol, 1998
- 3  **FliN** , together with  **FliM** , account for most of the C ring.  Ref: 8757287 J Mol Biol, 1996
- The flagellar switch apparatus is a complex macromolecular structure composed of at least three gene products, FliG,  **FliM** , and  **FliN** .  Ref: 1400175 J Bacteriol, 1992
- The transitions between counterclockwise and clockwise rotation are controlled by a switch complex composed of three proteins FliG,  **FliM** , and  **FliN**  and located at the base of the flagellar motor.  Ref: 8415608 Proc Natl Acad Sci U S A, 1993
- wild type  **FliN**  can supplement the  **FliN**  domain of the fusion protein without displacing the  **FliM**  domain.  Ref: 8755888 J Bacteriol, 1996
- We have examined basal body preparations from non motile or non chemotactic fliG,  **fliM**  and  **fliN**  mutant strains by electron microscopy and immunoblot gel analysis.  Ref: 7650739 J Mol Biol, 1995
- Phylogenetic analysis indicates that the polar and lateral FliG,  **FliM** , and  **FliN**  switch proteins are closely related and most likely arose as a gene duplication event.  Ref: 11948156 J Bacteriol, 2002
- wild type  **FliM**  and  **FliN**  together have no major positive or negative effect on the function of the fusion protein.  Ref: 8755888 J Bacteriol, 1996
- In this work, the genes that encode the  **FliM**  and  **FliN**  proteins of Rhodobacter sphaeroides were characterized.  Ref: 9683497 J Bacteriol, 1998
- the C ring complex may contain  **FliM**  and  **FliN** .  Ref: 8308888 J Mol Biol, 1994
- Among the many proteins needed for assembly and function of bacterial flagella, FliG,  **FliM** , and  **FliN**  have attracted special attention because mutant phenotypes suggest that they are needed not only for flagellar assembly but also for torque generation and for controlling the direction of motor rotation.  Ref: 8550421 J Bacteriol, 1996
- The FliG,  **FliM** , and  **FliN**  proteins of the bacterial flagellar motor are believed to interact with one another to form the switch complex, which in turn is thought to interact with one of the chemotaxis proteins, CheY.  Ref: 9356251 J Mol Biol, 1997
- The flagellar motor switch complex, consisting of the three proteins FliG,  **FliM** , and  **FliN** , plays a central role in bacterial motility and chemotaxis.  Ref: 10809678 J Bacteriol, 2000
- These basal bodies lacked C rings but had intact M rings, suggesting that  **FliM**  and  **FliN**  are part of the C ring but not a detectable part of the M ring.  Ref: 11591685 J Bacteriol, 2001
- The pseudorevertant, in contrast, synthesized a protein with the predicted size for the  **FliM**   **FliN**  fusion protein and no detectable  **FliM**  or  **FliN** .  Ref: 8755888 J Bacteriol, 1996
- The flagellar switch proteins FliG,  **FliM** , and  **FliN**  of Salmonella typhimurium were overproduced in Escherichia coli and partially purified in soluble form.  Ref: 8206846 J Bacteriol, 1994
- Incubation of basal bodies at pH 2.5 removed FliG,  **FliM** , and  **FliN**  but not FliF.  Ref: 11591685 J Bacteriol, 2001
- A putative complex of the three switch proteins, FliG,  **FliM**  and  **FliN**  appears to be directly involved in torque generation and control of direction of rotation.  Ref: 8308888 J Mol Biol, 1994
- We have determined the phenotype associated with each mutant protein, the ability of each mutant protein to interfere with the motility of wild type cells, and the effect of additional FliG and  **FliN**  on the function of selected  **FliM**  mutant proteins.  Ref: 8955386 J Bacteriol, 1996
- Minicell analysis demonstrated that, as expected, the parental mutant synthesized an essentially full length  **FliM**  protein but no detectable  **FliN** .  Ref: 8755888 J Bacteriol, 1996
- The  **fliM**  and  **fliN**  mutants are immotile at normal expression levels but become motile when the mutant proteins and or other, evidently interacting flagellar proteins are overexpressed.  Ref: 8550421 J Bacteriol, 1996
- FliG,  **FliM** , and  **FliN** , key proteins for torque generation, are located in two rings.  Ref: 10468575 Proc Natl Acad Sci U S A, 1999
- We have also examined the ability of various  **FliM**  deletion and truncation mutant proteins to bind to  **FliN** , FliG, and CheY P.  Ref: 9356251 J Mol Biol, 1997
- The switch is a complex apparatus composed of at least three proteins FliG,  **FliM**  and  **FliN** .  Ref: 8224881 Gene, 1993
- Complexes from the  **FliM**   **FliN**  fusion mutant gave a strong signal for FliG but no signal for either FIiM or  **FliN** .  Ref: 8755888 J Bacteriol, 1996
- These are MotA, MotB, FliG,  **FliM**  and  **FliN** .  Ref: 8757288 J Mol Biol, 1996
- Both  **FliM**  and  **FliN**  possess charged domains not found in proton type homologs.  Ref: 10648530 J Bacteriol, 2000
- Immunoblotting of minicells with antibodies against  **FliM**  and  **FliN**  confirmed the identities of these various proteins.  Ref: 8755888 J Bacteriol, 1996
- In order to elucidate the process of assembly of cytoplasmic substructures, the membrane localization of each component of the switch complex FliG,  **FliM** , and  **FliN**  in various nonflagellated mutants was examined by immunoblotting.  Ref: 9006037 J Bacteriol, 1997
- Analysis of a  **FliM**   **FliN**  flagellar switch fusion mutant of Salmonella typhimurium.  Ref: 8755888 J Bacteriol, 1996
- The switch complex is composed of three proteins, FliG,  **FliM** , and  **FliN** .  Ref: 9727015 J Biol Chem, 1998
- FliG,  **FliM** , and  **FliN** .  Ref: 9774535 J Struct Biol, 1998
- Flagella purified from Salmonella enterica serovar Typhimurium contain FliG,  **FliM** , and  **FliN** , cytoplasmic proteins that are important in torque generation and switching, and FliF, a transmembrane structural protein.  Ref: 11591685 J Bacteriol, 2001
- We report the sequence of 3978 bp of the Agrobacterium tumefaciens chromosome which contains a putative operon encoding the homologues of the transmembrane proton channel protein MotA, and the flagellar switch proteins  **FliM** ,  **FliN**  and FliG.  Ref: 9161425 Gene, 1997
- a moderately strong signal for the  **FliM**   **FliN**  fusion protein was seen with the anti  **FliM**  antibody, and a weaker signal was seen with the anti  **FliN**  antibody.  Ref: 8755888 J Bacteriol, 1996
